# Evaluation of the CropSyst Model during Wheat-Maize Rotations on the North China Plain for Identifying Soil Evaporation Losses

**DOI:** 10.3389/fpls.2017.01667

**Published:** 2017-09-29

**Authors:** Muhammad Umair, Yanjun Shen, Yongqing Qi, Yucui Zhang, Ayesha Ahmad, Hongwei Pei, Meiying Liu

**Affiliations:** ^1^Key Laboratory of Agricultural Water Resources and Hebei Key Laboratory of Water-Saving Agriculture, Center for Agricultural Resources Research, Institute of Genetics and Developmental Biology, Chinese Academy of Sciences, Shijiazhuang, China; ^2^International College, University of Chinese Academy of Sciences, Beijing, China; ^3^Climate Change Research Center, Institute of Atmospheric Physics, Chinese Academy of Sciences, Beijing, China; ^4^College of Energy and Environmental Engineering, Hebei University of Architecture, Zhangjiakou, China

**Keywords:** North China Plain, CropSyst, evaporation losses, crop rotation, model evaluation

## Abstract

The North China Plain (NCP) is a major grain production zone that plays a critical role in ensuring China's food supply. Irrigation is commonly used during grain production; however, the high annual water deficit [precipitation (P) minus evapotranspiration (ET)] in typical irrigated cropland does not support double cropping systems (such as maize and wheat) and this has resulted in the steep decline in the water table (~0.8 m year^−1^ at the Luancheng station) that has taken place since the 1970s. The current study aimed to adapt and check the ability of the CropSyst model (Suite-4) to simulate actual evapotranspiration (ET_a_), biomass, and grain yield, and to identify major evaporation (E) losses from winter wheat (WW) and summer maize (SM) rotations. Field experiments were conducted at the Luancheng Agro-ecosystem station, NCP, in 2010–2011 to 2012–2013. The CropSyst model was calibrated on wheat/maize (from weekly leaf area/biomass data available for 2012–2013) and validated onto measured ET_a_, biomass, and grain yield at the experimental station from 2010–2011 to 2011–2012, by using model calibration parameters. The revalidation was performed with the ET_a_, biomass, grain yield, and simulated ET_a_ partition for 2008–2009 WW [ET_a_ partition was measured by the Micro-lysimeter (MLM) and isotopes approach available for this year]. For the WW crop, E was 30% of total ET_a_; but from 2010–11 to 2013, the annual average E was ~40% of ET_a_ for the WW and SM rotation. Furthermore, the WW and SM rotation from 2010–2011 to 2012–2013 was divided into three growth periods; (i) pre-sowing irrigation (PSI; sowing at field capacity) to emergence period (EP), (ii) EP to canopy cover period (CC) and (iii) CC to harvesting period (HP), and E from each growth period was ~10, 60, and 30%, respectively. In general, error statistics such as RMSE, Willmott's *d*, and NRMSE in the model evaluation for wheat ET_a_ (maize ET_a_) were 38.3 mm, 0.81, and 9.24% (31.74 mm, 0.73, and 11.89%); for wheat biomass (maize biomass) they were 1.25 Mg ha^−1^, 0.83, and 9.64% (0.78 Mg ha^−1^, 0.96, and 7.96%); and for wheat grain yield (maize grain yield) they were 0.65 Mg ha^−1^, 0.82, and 9.87% (0.2 Mg ha^−1^, 0.99, and 3.79%). The results showed that CropSyst is a valid model that can be use with a reliable degree of accuracy for optimizing WW and SM grain yield production and water requirement on the NCP.

## Introduction

The North China Plain (NCP) is recognized as the “breadbasket of China.” It accounted for 25% of wheat and 18% of corn production from 2002 to 2011, and continues to play a major role in ensuring national food grain security. The plain occupies 8% of global arable land (2008) (FAO, 2010) and is home to 20% of the world's population (1.4 billion in 2010) (United Nations, 2012).

**Figure 1:**
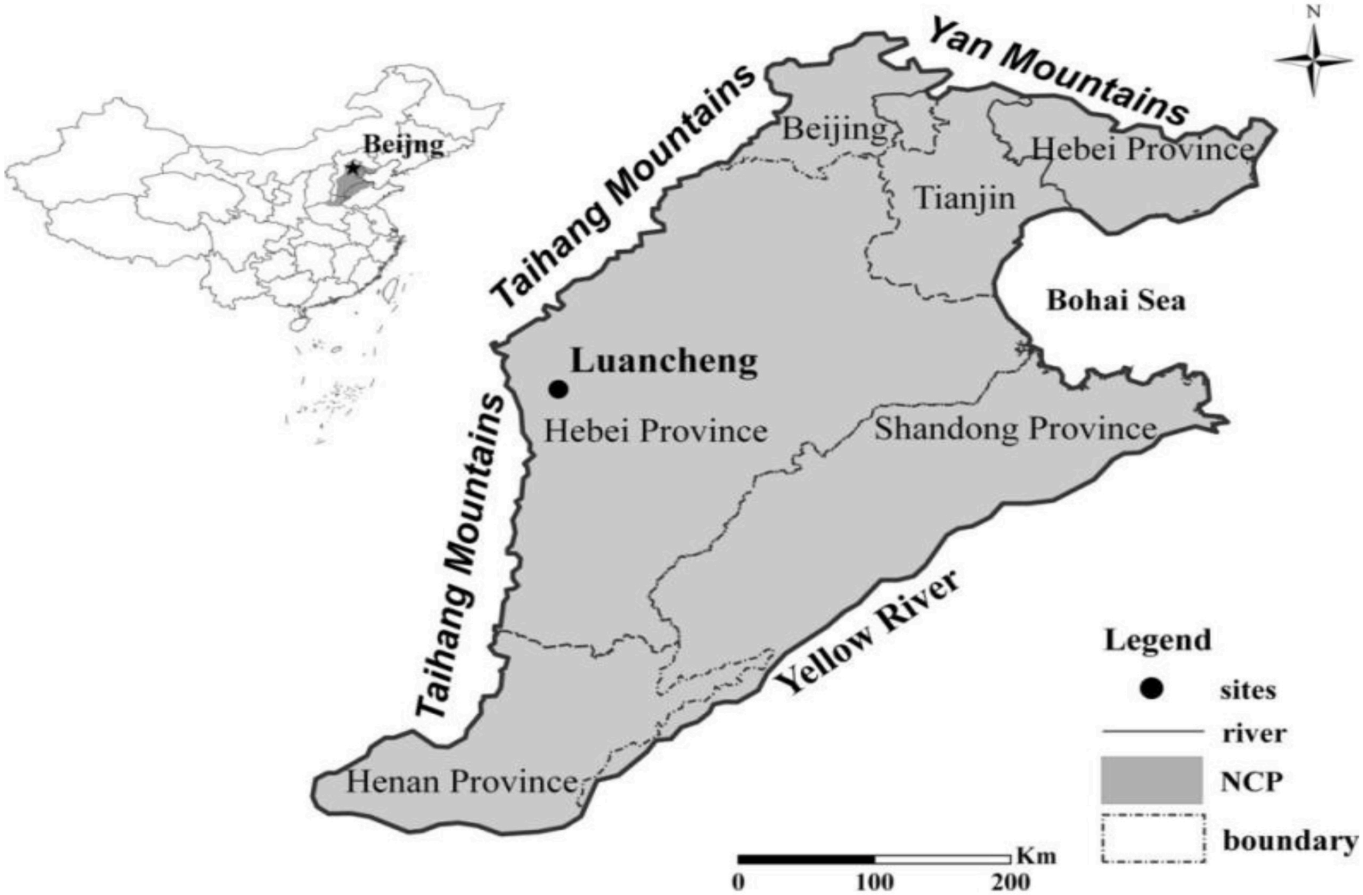
Location of Luancheng Agro-Ecosystem Experimental Station (LAEES) in the North China Plain.

Irrigation has played an integral role in the almost 8-fold increase in grain yield in the NCP from 0.64 t ha^−1^ in 1950 to ~5.00 t ha^−1^ in 2009 (Zhou et al., 2007). Grain yield relies largely on irrigation because rainfall (400–600 mm) and timing (mostly during the summer monsoon) are insufficient to support a double crop rotation such as maize and wheat. Irrigation is the most common water source throughout Hebei province in the Piedmont part of Mountain Taihang (Figure 1), with supplies originating from groundwater (~75% of the total irrigated land) and from surface water reservoirs (~25% of total irrigated land) (Liu et al., 2010; Sun et al., 2010). According to monitoring and model analyses in the Piedmont region, since the 1970s, large water deficits have been offset by high levels of groundwater withdrawal, resulting in a steep decline in water table levels [for example, ~0.8 m year^−1^ at the Luancheng Agro-Ecosystem Experimental Station (LAEES)]. At LAEES–the most representative farmland on the NCP–there has been a 35 m decrease in groundwater over the past 30 years, while overall in the NCP, there has been a 10 m decrease over the last 10 years. International agencies agree with the assessment of the declining water table in this region (Moench et al., 2003; Varley, 2005; Yuanxi, 2007).

Mean annual water deficit (P-ET) in the typical irrigated cropland in NCP is 230 mm for groundwater irrigated land. The deficit mainly occurs in the winter wheat (WW) season and ranges from 160 to 410 mm at LAEES (Shen et al., 2013). Long-term field experiments from 1987 to 2015, including 28 WW growing seasons in LAEES showed that, during that time, atmospheric evaporation demand (ET_0_) increased and seasonal rainfall decreased. Although yield continuously increased from 1987 to 2015 under irrigated conditions, the yield of WW under rain-fed conditions has decreased recently as compared with that during the 2000s, due to the higher ET_0_ and lower levels of seasonal rainfall (Zhang et al., 2017).

Climate change studies on the NCP indicate that this situation will further worsen with the increase in agricultural water demand and the predicted decline in water resources (Tao et al., 2003, 2005; Falloon and Betts, 2010). This position is the outcome of over irrigation by NCP farmers in their attempts to achieve high yields, especially during the wheat-growing season (Yang et al., 2002; Zhang et al., 2002). Water-use efficiency is still low due to poor irrigation management practices such as flood irrigation (Wang et al., 2002; Deng et al., 2006; Shao et al., 2009). In view of the above situation, there is an urgent need for alternative strategies that ensure sustainable application of small amount groundwater at site-specific and regional levels (Qadir et al., 2003).

Water usage is usually described as evapotranspiration (ET) over the entire crop growing period. Accurate monitoring or estimation of ET, as well as the biomass formation from cropland, are essential for estimating water use efficiency (WUE) (Todd et al., 2000; Shen et al., 2004; Shao et al., 2010). A variety of approaches have been used to monitor ET, including a weighing lysimeter (Liu et al., 2002; Castellví and Snyder, 2010), eddy covariance systems (Zhou and Zhou, 2009; Scott, 2010), and water balance modeling (Wilson et al., 2001; Sun et al., 2010). Several studies have been conducted using pots and field experiments to assess soil evaporation losses and to quantify the exact quantity of water applied to WW; the yield results were then compared with dissimilar water treatments on the NCP. For example, Liu et al. (2002) determined soil E, by using a micro-lysimeter and ET by employing a large scale lysimeter, and reported that E on WW and summer maize (SM) achieved levels of 30.3 and 29.7% of the total ET, respectively. Zhang et al. (2011) used a stable isotope mixing model with micro-lysimeter E measurements and eddy covariance (EC) evapotranspiration estimates to evapotranspiration partition and suggested that E from the soil surface during WW seasons took up to 30% of the total water consumption. Thus, on this plain with its severe water deficits, water saving techniques for reducing soil E are important management practices.

The present study was designed to calibrate and extensively validate the CropSyst model for ET_a_, biomass, and grain yield (2010–2013) under conditions of full irrigation on a WW–SM rotation. The second aim was to revalidate the ET_a_, biomass, grain yield, and ET_a_ partitioning into evaporation and transpiration on WW (2008–09). The final aim was to partition the ET_a_ from 2010 to 2013 to identify the main soil E losses.

## Materials and methods

### Study site, field experiments, and measurements

The field experiments were conducted on typical WW and SM crop rotations at LAEES Chinese Ecological Research Network, Luancheng County, Shijiazhuang, Hebei Province, China as shown in Figure 1. Shen et al. (2002) note that it is a prominent productive area yielding about 13,500 kg ha^−1^/y under a WW–SM rotation. The WW crop period from early October to the following mid-June is about 247 days, while the SM crop period from mid-June to late September is about 107 days. The area has a moderate dry monsoon climate, with a mean annual global radiation of 524 kJ/cm^2^ and a mean annual temperature of 12.2°C. Most (60–80%) of the mean annual rainfall of 481 mm occurs during the SM season (June–September), while relatively little falls during March–May at a time when WW grows rapidly, as shown in Table 1. Rainfall does not fulfill the water requirement for WW growth, particularly throughout the windy, dry spring.

**Table 1:** Rainfall throughout the wheat and maize cropping periods from 2010–2011 to 2012–2013, at Luancheng Agro-Ecosystem Experimental Station (LAEES).

**Year**	**Months**												**Total (unit: mm)**
	**Oct**	**Nov**	**Dec**	**Jan**	**Feb**	**Mar**	**Apr**	**May**	**Jun**	**Jul**	**Aug**	**Sep**	
2010–2011	4.4	0	1.6	0	8.7	0	0.9	30.7	44.3	116	80.1	77.1	363.8
2011–2012	15.5	30	0.2	0.1	0	6.2	18.7	0	61.9	213.2	125.2	83.2	554.2
2012–2013	1.5	22.5	4.4	4.6	9.6	0.5	29.2	14.7	84.5	133.9	173.7	61.8	540.9

The experimental plots (5 × 10 m) were created and divided by concrete walls. According to the FAO (Food and Agricultural Organization) the walls extended 1.5 m under the soil surface and 24.5 cm thick. The fields had been mulched with straw from the WW and SM for ~20 years and rotary tillage was practiced (with a tilling depth of about 10 cm) to mix the broken straw with the top soil; it is termed a reduce tillage practice because the tilling depth is shallower than that of normal traditional tillage at around 25–30 cm. The WW variety Kenong No.199 was sown at a rate of 187 kg ha^−1^ in 25 cm wide rows. The SM variety Zhengdan No.958 was sown by hand at a rate of 53 kg ha^−1^ into 50 cm wide rows. Fertilization schedules included di-ammonium phosphate, applied before wheat sowing at a rate of ~600 kg/ha; then, supplementary urea was applied twice with irrigation at the beginning of April (August) 300 kg/ha (600 kg/ha) during the jointing stages of WW (SM), respectively. Generally, farmers apply manure every 2–3 years at a rate of around 100 kg N/ha. The soil type is silt loam, with a field capacity of ~35% (Sun et al., 2007; Shen et al., 2011). Soil organic matter content was 1.8% in the top 20 cm layer at the experimental site. Soil parameters and features are shown in Table 2.

**Table 2:** Soil attributes at the experimental site at Luancheng Agro-Ecosystem Experimental Station (LAEES).

**Layer**	**Depth (cm)**	**Thickness (m)**	**Sand**	**Clay**	**Silt**	**Bulk Density (g/cm^3^)**	**FC (m^3^/m^3^)**	**kSat (m/day)**
1	0–20	0.1	15.00	20.00	65.00	1.300	0.280	0.2112
2	20	0.1	15.00	20.00	65.00	1.300	0.280	0.2112
3	20–35	0.15	20.00	20.00	60.00	1.400	0.280	0.2112
4	40	0.05	15.00	25.00	60.00	1.460	0.280	0.2112
5	60	0.2	15.00	25.00	60.00	1.460	0.280	0.2112
6	80	0.2	15.00	25.00	60.00	1.460	0.280	0.2112
7	100	0.2	15.00	25.00	60.00	1.47	0.291	0.17
8	120	0.2	15.00	25.00	60.00	1.500	0.322	0.0288
9	140	0.2	50.00	45.00	5.00	1.540	0.323	0.0288
10	160	0.2	50.00	45.00	5.00	1.540	0.323	0.0288
11	180	0.2	50.00	45.00	5.00	1.540	0.323	0.0288

A low pressure water transportation system outlet connected to a plastic pipe was used to irrigate each plot and water use was recorded by a water meter. Normally, 3–4 irrigation applications of 60–80 mm each were given during the WW growing season and 1–2 irrigations were applied for SM, depending on rainfall. Levels of irrigation and their timings are shown in Table 3. Leaf area index (LAI) was estimated by randomly measuring the leaf area of plant samples. Each sample included 10 wheat plants or 3 maize plants. The sum of the leaf area was scaled to a unit area according to plant density and then used to calculate the leaf area index. Plant density was observed simultaneously. Grain yield measurement was taken in the middle of each plot (area of 3 × 8 m), and a 1,000-kernel weight was determined from the harvested grains. For dry matter, 10 plants were measured after 48 h of oven drying at 65°C.

**Table 3:** Management measures during winter wheat and summer maize seasons from 2010–2011 to 2012–2013, at Luancheng Agro-Ecosystem Experimental Station (LAEES).

**Management measures**	**Wheat**	**Corn**	**Wheat**	**Corn**	**Wheat**	**Corn**
Sowing date	11/10/2010	15/06/2011	07/10/2011	11/06/2012	10/10/2012	12/06/2013
Harvest date	13/06/2011	02/10/2011	09/06/2012	01/10/2012	11/06/2013	30/09/2013
Seed density (seeds/ha)	3,200,000	600,00	3,200,000	60,000	3,200,000	60,000
Line spacing (cm)	20	60	20	60	20	60
1st Irrigation date	23/11/2010	16/06/2011	16/04/2012	21/06/2012	02/12/2012	
Irrigation (cm)	8	9.4	12.2	7.1	0.368	
2nd Irrigation date	13/04/2011	13/07/2011	03/05/2012		06/04/2013	
Irrigation (cm)	6	13	8.5		12.85	
3rd Irrigation date	02/05/2011		31/05/2012		07/05/2013	
Irrigation (cm)	8.4		12.3		14.31	
4th Irrigation date	26/05/2011				2013/05/20	
Irrigation (cm)	6.8				9.41	
Total Irrigation (cm)	29.2	22.4	33	7.1	36.46	

**Figure 2:**
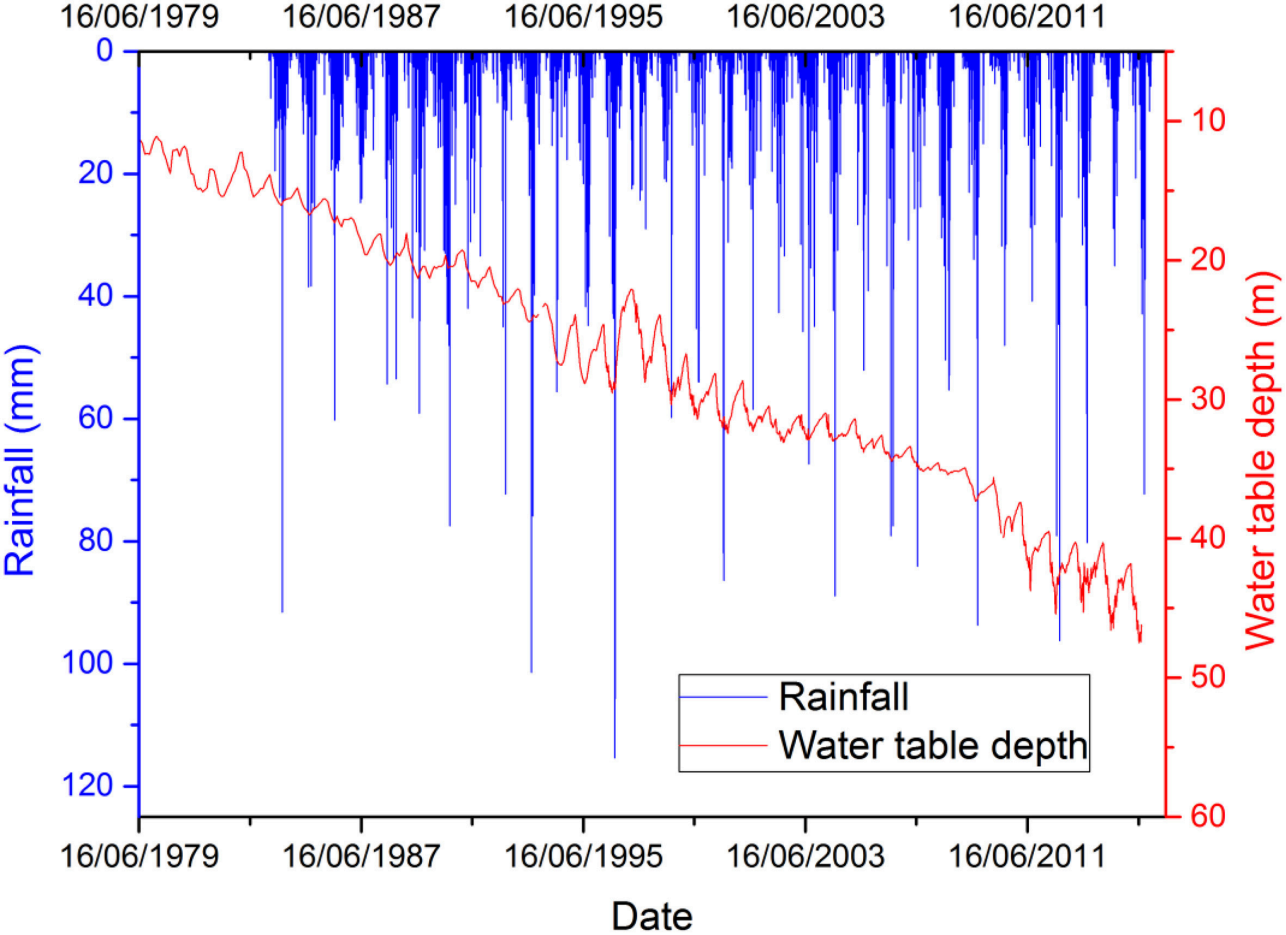
Ground water dynamic changes 1974–2016 at Luancheng Agro-Ecosystem Experimental Station (LAEES).

ET measured by an eddy covariance (EC) system consisted of a CSAT3 sonic-anemometer and a LI7500 H2O/CO2 gas analyzer (Campbell Scientific, Inc. USA) at 3 m above ground level. Latent heat flux was measured every 30 min. The water table declined rapidly from 1975 to 2016 with a drawdown rate of about 0.8 m per year as shown in Figure 2.

The water table increased a little after rainfall, but a dramatic decrease was noted at the start of each successive irrigation period. Weather data was recorded on a daily basis, including rainfall, maximum/minimum temperature, relative humidity, dew point, and wind speed throughout the experimental period; this was obtained from the meteorological station at LAEES. The Angstrom equation was used for the calculation of daily solar radiation from the number of sunshine hours (Jones, 1992). Field data was collected from 2010–2011 to 2012–2013 at LAEES and thereafter used to calibrate and validate the CropSyst Model. Management measures during WW and SM growing seasons from 2010–2011 to 2012–2013 are shown in Table 3.

### CropSyst model

The ability to simulate crop rotations is important in the analysis of cropping systems. Models used in rotation configuration belong to the CERES and CROPGRO families; these are all situated under the umbrella of DSSAT (Jones et al., 1998). Jones et al. (2001) report that DSSAT access has been slow to adopt a more generic simulation platform, hence users tend to merge these models to simulate crop rotation. Efficient and simple multi-crop simulation takes place in the EPIC model for the analysis of cropping systems and rotations (Willmott, 1982). CropSyst was designed to depict the EPIC concept for the analysis of crop growth, including a more process-oriented approach to simulate crop rotation and its interaction with the surrounding environment and management (Stockle et al., 1994; Stöckle et al., 2003). CropSyst is a cropping system model that is designed to simulate a range of management and weather scenarios. This model is suitable for use in the study of soil, water budget, nitrogen budget, and weather variables. The predictive capability of a model determines its reliability, application, and performance evaluation for its use as a research tool. Therefore, in this study, we used the CropSyst model to simulate growth yield and water use of a widely grown rotation of SM and WW in NCP. The results of this study should offer valuable information about agricultural water management and help assess approaches that involve reducing water usage while maintaining crop productivity.

### Input data

CropSyst (Suite-4) was used to model crop rotations and crop productivity in reaction to soil, management, and weather. Crop growth stages were simulated based on thermal time demands to different developmental stages. CropSyst used five input files for simulation: (i) simulation control (a combination of different input files such as start and ending days, crop rotation, initialization parameters, simulation of soil salinity, erosion, CO_2_, and nitrogen effectuates on crop development, and a choice of soil runoff and water distribution models) based on field experiments; (ii) position file [latitude, weather file, rainfall volume parameters, ET model selection Penman-Monteith (PM) or Priestley-Taylor (PT) on automatic mode] obtained from the weather station of the experimental site; (iii) soil file (soil type, pH, wilting point, bulk density, field capacity, cation exchange capacity, and hydraulic conductivity) based on existing field soil properties; (iv) management file (irrigation, fertilization, tillage, residue, conservation, and harvesting) based on field management operation; and (v) crop file (emergence, thermal time accumulation, transpiration, attain growth, canopy growth, phenology, vernalization, photo-period, senescence, dormancy, and harvest). Detailed data of weekly leaf area, biomass, and grain yield were measured, and harvest index was determined during the experiment, that used for crop file preparation. The parameters used for the CropSyst model were measured or estimated using experimental data; some of this information was based on field experience, and other parameters used the default values given in the model, regardless of the year (Table 4).

**Table 4:** Parameters used in the CropSyst model to simulate winter wheat and summer maize cropping.

**Parameter**	**Wheat**		**Maize**	
	**Value**	**Source**	**Value**	**Source**
**PHENOLOGY**				
Degree-days emergence (°C^−d^)	100	M	100	M
Degree-days begin flowering (°C^−d^)	1,234	M	1,091	M
Degree-days begin grain filling (°C^−d^)	1,337	M	1,232	M
Degree-days physiological maturity (°C^−d^)	1,953	M	1,711	M
Base temperature (°C)	0	L	8	L
Cutoff temperature (°C)	25	L	30	L
**MORPHOLOGY**				
Maximum root depth (m)	2	M	2	M
Maximum LAI (m^2^ m^−2^)	5	M	5	M
Specific leaf area (m^2^ kg^−1^)	22	C	22	C
Leaf duration (°C^−d^)	1,000	M	850	M
Leaf duration sensitivity to stress	2	C	1	C
Extinction coefficient for solar radiation	0.5	L	0.5	L
ET crop coefficient at full canopy	1.35	C	0.8	C
**GROWTH**				
Maximum water uptake rate (mm d^−1^)	8	L	12	L
Critical leaf water potential (J kg^−1^)	−1,300	D	−1,200	D
Wilting leaf water potential (J kg^−1^)	−2,300	D	−2,000	D
Above ground biomass-transpiration coefficient (kPa kg m^−3^)	5.7	L	8.5	L
Unstressed harvest index	0.49	M,E	0.58	M,E
Fertility Stress	None	N	None	N

*C, Calibrated; D, default; M, measured; L, literature; E, local experience; N, not consider*.

## Model calibration, validation, and evaluation

### Parameters

The CropSyst model allows calibration of cultivar sensitive parameters for crops. A daily weather database, experimental phenologic stages, weekly biomass, weekly green leaf area indices, and biomass and grain yield at maturity were input for each growing season to calibrate phenology, morphology, and growth parameters (Table 4) for the study location.

### Calibration of the CropSyst model

Calibration of the CropSyst model was performed using values observed from the experiment during 2012–2013 of WW and SM on full irrigation, for ET_a_, biomass, and grain yield. Differences between the simulated model and the measured data were minimized by applying a trial and error approach. A detailed data set of weekly leaf area and biomass was available for 2012–2013, so this was selected for calibration.

### Validation of the Cropsyst model

Validation was performed by equating field observed data with simulated results and it is a significant measure in model assessment (Power, 1993; Addiscott et al., 1995). ET_a_, biomass, and grain yield were considered as the verification parameters for the CropSyst model. Crop parameters obtained from the model calibration were used in the validation. The independent data set was used to test the model's performance from 2010 to 2012.

### Revalidation of the CropSyst model

A revalidation of the model depends upon its successful calibration based on field experimental data, and the accurate estimation of the specific model's coefficients in a given environment. Eitzinger et al. (2003) revalidated the CERES-Wheat model using the grain yield observed over 9 years (1985–1993) at the experimental site, and compared it with model outputs. Iqbal et al. (2014) revalidated the AquaCrop model for wheat on the NCP under deficit irrigation from field experiments to regional yield simulation. In the current study for the CropSyst evaluation, we used ET_a_, biomass, grain yield, and ET_a_ partition data measured at the Luancheng experimental site for WW (2008–2009) using well-water irrigation. Small experiment plots (5 × 10 m) were constructed with concrete walls to avoid soil water recharge, according to stipulations adjusted by the FAO. Plot experiments have been in operation for approximately 15 years. The well-watered plots were irrigated to maintain ≥85% of field capacity. Three irrigation applications totaling 230 mm were given in April to May 2009. Soil volumetric moisture content was measured using a neutron probe (IH-II, Institute of Hydrology, Wallingford, UK) at 10 cm intervals between a depth of 0 and 180 cm. Depth of the water table was measured with a water level logger (Hobo U20-001-01, Onset Corp., Bourne, MA). Vapor pressure deficit was calculated from temperature and relative humidity data that was measured with a temperature-relative humidity data logger at 0.1, 3, and 10 m. Soil temperatures at 0.05 m were measured with a digital thermometer. ET was measured by an EC system composed of a CSAT3 sonic-anemometer and a LI7500 H_2_O/CO_2_ gas analyzer (Campbell Scientific, Inc.) at 3 m above ground level. Latent heat flux was measured every 30 min. Evaporation (E) data was measured using two micro-lysimeter systems (MLS) (Shen et al., 2002; Sun et al., 2006). Stable isotopes are excellent tracers of soil water cycling because of the isotopic fractionation imparted by E. Isotope composition of soil water, groundwater, plant-stem water, rainfall, and atmospheric water vapor were analyzed over one growing season. ET_a_ and its partitioning was performed by combined isotopic and micro-meteorologic approaches in an irrigated WW field; a more detail description of this experiment can be found in a related paper, Zhang et al. (2011). This season was chosen for revalidation because transpiration (T) and E data, collected by a micro-lysimeter and isotopes, was available.

### Model evaluation

Since no individual criterion can accurately gauge the accuracy of a simulation model, combine statistical indexes are broadly applied to judge model performance (Caton et al., 1999; Kobayashi and Salam, 2000; Gauch et al., 2003). Correspondence between the observed and simulated values were measured by adopting six statistics: RMSE (root mean square error), NRMSE (normalized root mean square error), MBE (mean bias error), MAE (mean absolute error), and IoA (index of agreement) (Willmott, 1982). Percentage differences were determined using the following equations, where Si indicates simulated values and Mi indicates measured values of all statistical indices.

(1) $$\text{RMSE}=\sqrt{\frac{1}{\text{n}}\sum {\left(\text{Si}-\text{Mi}\right)}^{2}}$$

(2) $$\text{NRMSE}=\sqrt{\sum \frac{{\left(\text{Si}-\text{Mi}\right)}^{2}}{\text{n}}}\times \frac{100}{\text{M}}$$

(3) $$\text{MAE}=\frac{1}{\text{n}}\sum \left|\text{Si}-\text{Mi}\right|$$

(4) $$\text{MBE}=\frac{1}{\text{n}}\left[\sum \text{Si}-\text{Mi}\right]$$

(5) $$\text{d}=1-\left[\frac{\sum {\left(\text{Si}-\text{Mi}\right)}^{2}}{\sum {\left(\left|\text{Si}-\bar{\text{M}}\right|+\left|\text{Mi}-\bar{\text{M}}\right|\right)}^{2}}\right]$$

(6) $$\text{Percent deviation}=\left(\text{Simulated}-\text{Measured}\right)\times \frac{100}{\text{Measured}}$$

The simulation results are viewed as excellent when an NRMSE of <10% is achieved, good if the NRMSE is >10% <20%, fair if the NRMSE is >20% <30%, and poor if the NRMSE is >30% (Jamieson et al., 1991). For IoA, a measure result of 1.0 indicates excellent correspondence between measured and simulated values. For RMSE, a result close to zero indicates better model performance. MBE reveals the duration of execution of the model. A positive MBE result gives the mean sum of overvaluation in the estimated values and vice versa. MAE measures the weighted average magnitude of the absolute errors.

## Results

### CropSyst model calibration

The CropSyst calibrated data set of WW (2012–2013) and SM (2013) is shown in Table 5 for ET_*a*_, biomass, and grain yield. In WW, the minimum (maximum) deviation in grain yield (ET_a_) was 4.76% (9.51%), respectively, while the deviation in biomass was 5.03%. For the SM crop, the minimum (maximum) deviation in ET_a_ (grain yield) was −0.11% (−0.51%), respectively, while biomass deviation was 0.27%. The calibration results show a reasonably close match between the measured values and those simulated by the model (Table 5).

**Table 5:** Measured vs. simulated results for calibrated and validated data sets of winter wheat and summer maize from 2010 to 2013 at Luancheng Agro-Ecosystem Experimental Station (LAEES).

**Year**		**Crop**	**Grain yield (Mg ha**^**−1**^**)**			**Biomass (Mg ha**^**−1**^**)**			**ET**_**a**_ **(mm)**		
			**Measured**	**Simulated**	**Deviation (%)**	**Measured**	**Simulated**	**Deviation (%)**	**Measured**	**Simulated**	**Deviation (%)**
Calibration Year	2012–2013	Winter wheat	6.27	6.57	4.76	12.81	13.45	5.03	401.78	440.00	9.51
	2013	Summer maize	4.61	4.58	−0.51	8.01	8.03	0.27	272.29	272.00	−0.11
Validation Year	2010–2011	Winter wheat	6.94	7.37	6.29	13.66	15.05	10.20	461.77	435.00	−5.80
	2011	Summer maize	6.07	6.06	−0.21	11.25	10.66	−5.25	222.80	269.65	21.03
	2011–2012	Winter wheat	6.49	5.50	−15.26	12.78	11.26	−11.90	379.85	427.00	12.41
	2012	Summer maize	5.50	5.15	−6.42	10.22	9.01	−11.92	305.76	277.00	−9.41

*ET_a_, Actual evapotranspiration*.

### CropSyst model validation

Considering the same parameters used in the calibration procedure, CropSyst was validated for 2010–2011 to 2011–2012, as shown in the second part of Table 5.

#### Actual evapotranspiration (ET_a_)

Table 5 shows validation of the CropSyst model for ET_a_ in the experimental years of 2010–2011 to 2011–2012. In the first year of validation, CropSyst simulated the largest deviation (21.03%) for SM (2011). This suggests the lowest drainage levels plus seasonal rainfall contributed to an overestimation of ET_a_, but produced a satisfactory grain yield and biomass. Other underestimated deviation of ET_a_ (−5.8%) in the WW (2010–2011) also prostrates to drainage; in contrast, grain yield and biomass were simulated by CropSyst to an acceptable level. For the validation year of 2011–2012, WW showed a positive deviation (12.41%) and SM under-estimated the deviation (−9.41) of ETa with the CropSyst model, but produced a satisfactory grain yield and biomass. Table 6 contains all the model evaluation criteria from 2010–2011 to 2012–2013, with RMSE (38.3 mm), MAE (37.38 mm), MBE (19.35 mm), d (0.81), and NMRSE (9.42%) for WW, while for SM the following were achieved: RMSE (31.74 mm), MAE (25.30 mm), MBE (5.94 mm), d (0.73), and NMRSE (11.42%). The model therefore gave an acceptable simulation of the value of ET_a_ for WW and SM in the arid-semiarid conditions of North China.

**Table 6:** Simulation error statistics of winter wheat and summer maize.

**Crops**	**Model output parameters**	**Mean**		**RMSE**	**NRMSE (%)**	**IoA**	**MAE**	**MBE**
		**Measured**	**Simulated**					
Winter Wheat	ET_a_ (mm)	414.47	434.00	38.30	9.24	0.81	37.38	19.53
	Biomass (Mg ha^−1^)	13.08	13.26	1.25	9.54	0.83	1.19	0.17
	Grain yield (Mg ha^−1^)	6.56	6.48	0.65	9.87	0.82	0.57	−0.08
Summer Maize	ET_a_ (mm)	266.95	272.88	31.74	11.89	0.73	25.30	5.94
	Biomass (Mg ha^−1^)	9.83	9.23	0.78	7.96	0.96	0.61	−0.60
	Grain yield (Mg ha^−1^)	5.39	5.26	0.20	3.79	0.99	0.13	−0.13

*RMSE, Root mean square error; NRMSE, normalized root mean square error; MBE, mean bias error; MAE, mean absolute error; IoA, index of agreement*.

#### Final aboveground biomass

Final above ground biomass validation of the CropSyst model is shown in Table 5. The highest negative deviations (−11.90%, −11.92%) were simulated for WW (2011–2012), and for SM (2012), respectively. This was due to severe water stress experienced during the cropping season. Another significant positive deviation (10.2%) was noted for the WW 2010–2011 seasons, where irrigation was omitted during the grain filling period, and this treatment received the lowest amount of irrigation. The additional amount of water obtained from rainfall could lead to an over estimation of the biomass. Overall statistical parameters for model evaluation in Table 6 show: RMSE (0.78 Mg ha^−1^), MAE (0.61 Mg ha^−1^), MBE (−0.60 Mg ha^−1^), d (0.96), and NMRSE (7.96%) of SM biomass and an RMSE (1.25 Mg ha^−1^), MAE (1.19 Mg ha^−1^), MBE (−0.17 Mg ha^−1^), d (0.83), and NMRSE (9.54%) of WW biomass, respectively. The results of the present study revealed that the CropSyst model effectively simulated the above ground biomass of WW and SM.

#### Grain yield

The validation year results in Table 5 show no significant deviation in grain yield for WW (6.29%) and SM (−0.21%) between the CropSyst measured and simulated values for the year 2010–2011. In the second year of validation (2011–2012), an underestimated deviation (−15.2%) was simulated for WW grain yield as this year was also relatively dry and the cropping season depended mainly upon irrigation, as already explained for biomass. Meanwhile, a −6.42% deviation was simulated for SM grain yield. Statistical assessment of the 3 years experiment with the CropSyst model showed that the RMSE (0.65 Mg ha^−1^), MAE (0.57 Mg ha^−1^), MBE (−0.08 Mg ha^−1^), d (0.82), and NMRSE (9.87%) of WW grain yield and the RMSE (0.2 Mg ha^−1^), MAE (0.13 Mg ha^−1^), MBE (−0.13 Mg ha^−1^), d (0.99), and NMRSE (3.79%) of SM grain yield, respectively, were within an acceptable range. These results suggest that CropSyst is a valid model for WW and SM grain yield simulation.

### Revalidation of the CropSyst model on winter wheat (2008–2009)

The results of revalidation in Table 7 show no significant deviation in grain yield (−2.8%), biomass (−1.81), and ET_a_ (4.12%); the measured and simulated variables are a close match. The results of this study suggested that the CropSyst model can be used with a considerable degree of accuracy to simulate grain yield, biomass, and ET_a_ of WW in the NCP.

**Table 7:** Measured vs. simulated results for revalidated data sets of winter wheat of 2008–2009 at Luancheng Agro-Ecosystem Experimental Station (LAEES).

**Year**	**Variables**	**Measured**	**Simulated**	**Deviation (%)**
2008–2009 Winter wheat	ET_a_ (mm)	413.00	430.00	4.12
	Biomass (Mg ha−1)	13.24	13.01	−1.81
	Grain yield (Mg ha−1)	6.35	6.17	−2.80

*ET_a_, Actual evapotranspiration*.

### ET_a_ partitioning into evaporation and transpiration for winter wheat 2008–2009

**Figure 3:**
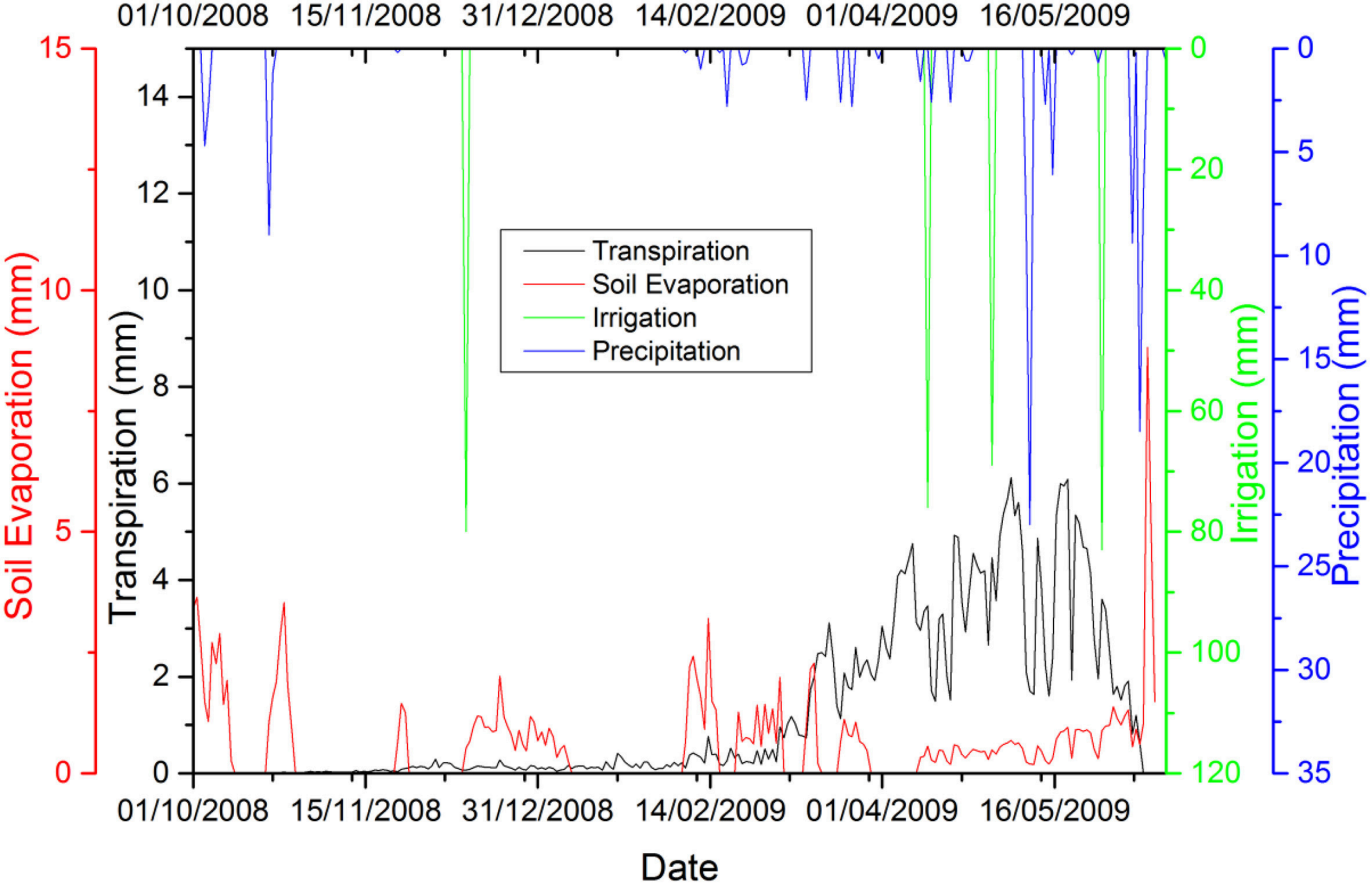
Total daily rainfall (including irrigation) and evapotranspiration partition during the winter wheat growing season of 2008–2009.

After successful revalidation of the CropSyst model for WW, simulated ET_a_ partitions were performed into E and T. The temporal trends in rainfall, irrigation, and simulated ET_a_ illustrate that ET_a_ increased substantially when rainfall or irrigation occurred (Figure 3). During growth periods for WW from planting to harvesting, simulated ET_a_ partitions of WW (2008–2009) into E and T show that the total ET_a_ was 430 mm, and E and T were 129 mm and 301 mm, respectively.

Furthermore, during the field experiment, T was measured by two methods, (i) E was measured using a micro-lysimeter (E(T)-MLS) and subtracted from ET_a_ as measured by EC; (ii) T was measured using the stable isotope method (E(T)-ISO). Complete isotopic data was collected on two dates representing the late stage of filling and the stage of wax ripeness [days of year (DOY) 138 and DOY149, respectively] and E data from MLS recordings available on the following days of year, as shown in Table 8. Observed T was 83% of total ET_a_ on DOY138 and 60% of ET_a_ on DOY149. The maximum percentage of T occurred during the filling stage; this was expected because this stage corresponds to a higher LAI and increasing biomass.

**Table 8:** Ratio of simulated transpiration.

**DOY**	**Data Source**	**Observed T/ET_a_**	**Simulated T/ET_a_**	**Deviation**
82	MLS	0.74	0.69	−6.59
86	MLS	0.81	0.79	−2.63
101	MLS	0.96	0.90	−6.76
116	MLS	0.84	0.90	6.97
120	MLS	0.86	0.90	5.03
123	MLS	0.84	0.90	7.32
128	MLS	0.85	0.90	5.66
136	MLS	0.94	0.89	−5.96
138	ISO	0.83	0.86	3.61
149	ISO	0.60	0.64	6.67
153	MLS	0.61	0.61	0.01

*MLS, micro-lysimeter; ISO, isotope method; EC, eddy covariance; DOY, days of year; T, transpiration; ET_a_, actual evapotranspiration*.

**Figure 4:**
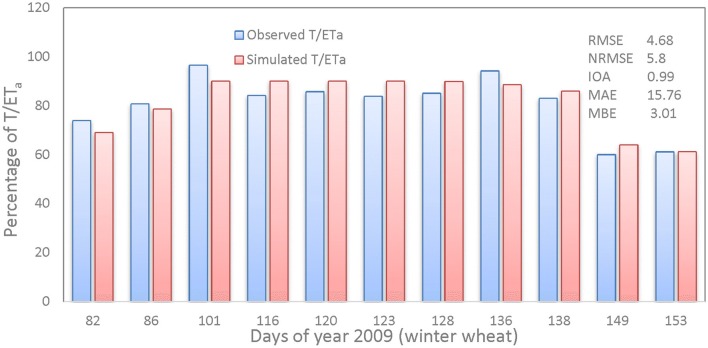
Measured vs. simulated percentage comparison of transpiration (T)/actual evapotranspiration (ET_a_).

Figure 4 shows the measured vs. simulated percentage comparison of T/ET_a_ and statistical assessment of the CropSyst model for the WW season of 2008–2009. The results of RMSE, MAE, MBE, d, and NRMSE were 4.68, 15.78, 3.01, 0.99, and 5.8% respectively, for WW grain yield.

**Figure 5:**
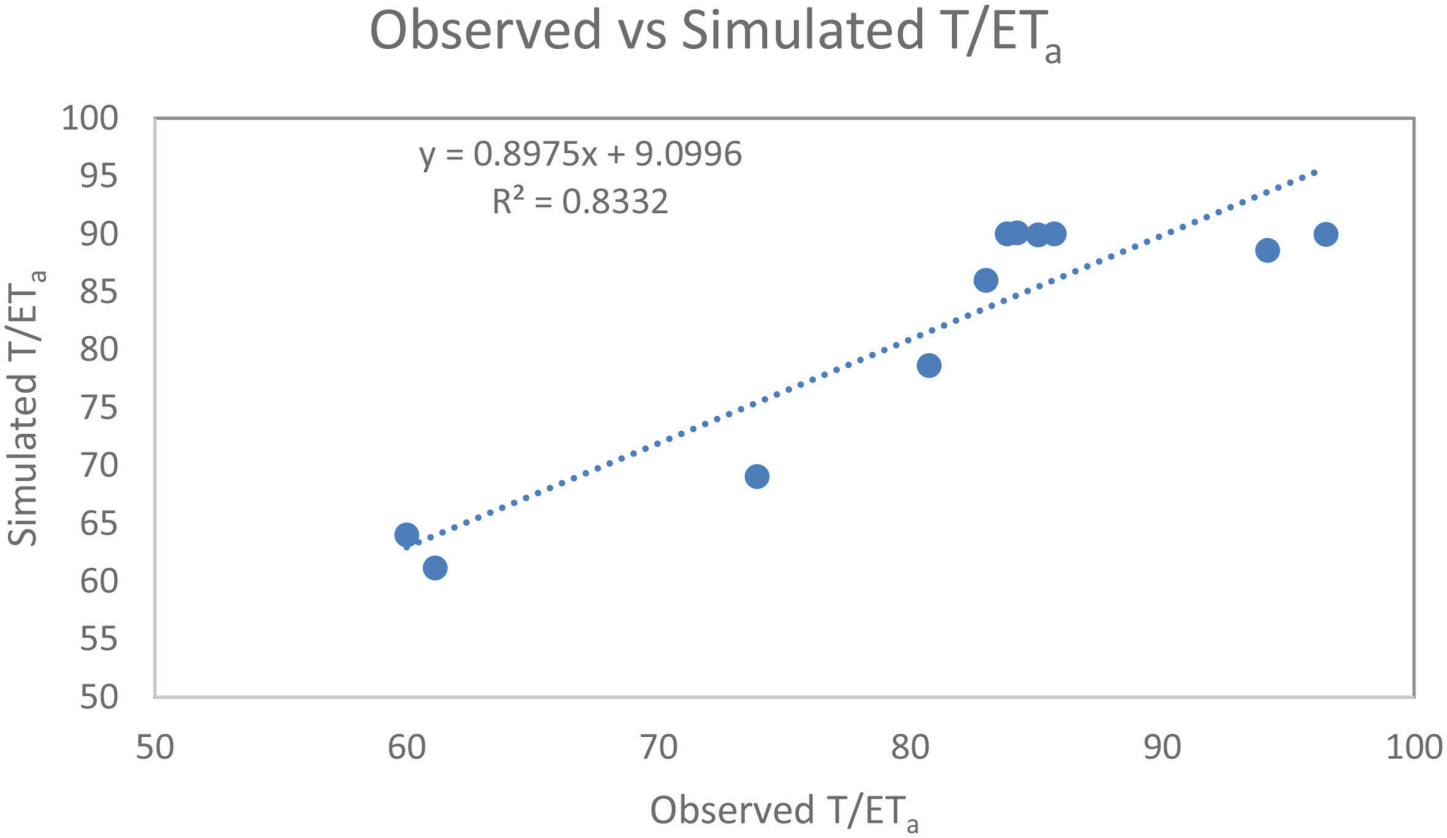
Relationship between the observed and simulated winter wheat transpiration (T)/actual evapotranspiration (ET_a_).

The straight-line equation and coefficient of determination shows that the model simulated T/ET_a_ with a high degree of reliability, having a regression value of 0.83, as shown in Figure 5.

### ET_a_ partition of wheat and maize crop rotation to identify evaporation losses

**Figure 6:**
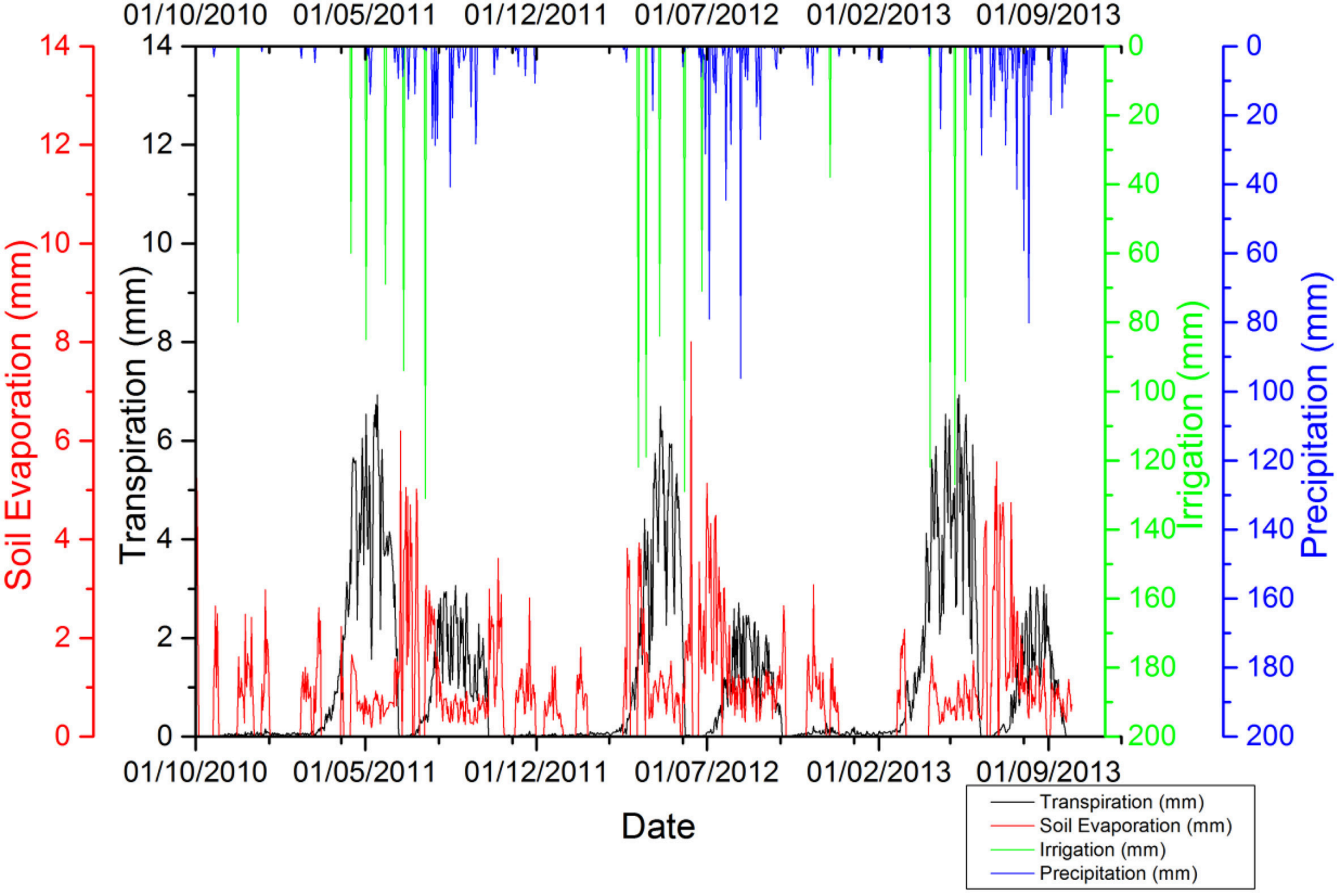
Simulated ET_a_ partition results of winter wheat and summer maize 2010–2011 to 2012–2013.

The simulated ET_a_ partitions into E and T of WW and SM from 2010 to 2013 are given in Figure 6 and show that the average value of total ET_a_, E, and T were 707, 282, and 425 mm, respectively. The temporal trend in rainfall, irrigation, and ET partition illustrates that ET_a_ increased substantially during rainfall or irrigation, (for example see Figure 6).

## Discussion

### Cropsyst model calibration

When compared to past studies, the CropSyst model calibration of WW and SM showed better results, i.e., Singh et al. (2008) simulated the highest deviation for wheat biomass, at −14.5% (underestimate) and Donatelli et al. (1997) simulated the highest deviation (0.96%) in maize grain yield.

### Cropsyst model validation

#### Actual evapotranspiration (ET_a_)

The large deviation in ET_a_ during the SM season (2011) was due to the high rainfall and low drainage. The negative (underestimated) deviation during the WW season (2010–2011) was also prone to drainage. During the next year of validation (2011–2012), WW (SM) were overestimated (underestimated) by the CropSyst model, meanwhile, both years produced a satisfactory grain yield and biomass. Based on field experiments, Sun et al. (2006) indicated that a full irrigation treatment had the highest amount of drainage, lowest water-use efficiency, and produced a lower yield. This could be the cause of the underestimation of ET_a_. Model evaluation criteria of ET_a_ for WW and SM from 2010–2011 to 2012–2013 fell within an acceptable range, as shown in Table 6. The RMSE of ET_a_ in WW and SM was within the excellent range and comparable with Fang et al. (2010), who simulated ET_a_ using RZWQM2 with a RMSE of 41.5 mm for a wheat-maize cropping system on the NCP. The CropSyst model calibrated ET_a_ more accurately with minimum deviation when compare to validation results.

#### Final aboveground biomass

The highest negative deviation of above ground biomass in WW (2011–2012) and SM (2012) was similar to grain yield deviation; this was due to water stress. A positive deviation was noted in WW biomass in 2010–2011; this was due to the grain filling stage being excluded from irrigation but being exposed to extra rainfall. A similar trend was observed in the CropSyst model validation for grain yield. Hsiao et al. (2009) presented a deviation between −0.4 and 21.9% for maize biomass simulation in the AquaCrop model. Table 6 shows the overall statistical parameters for model evaluation. Values of RMSE (0.78 Mg ha^−1^), MAE (0.61 Mg ha^−1^), MBE (−0.60 Mg ha^−1^), d (0.96), and NMRSE (7.96%) for SM were comparable with those results obtained by other studies (Stockle et al., 1997). WW results for RMSE (1.25 Mg ha^−1^), MAE (1.19 Mg ha^−1^), MBE (−0.17 Mg ha^−1^), d (0.83), and NMRSE (9.54%) were comparable with Wang et al. (2006) and Singh et al. (2008).

Lu and Fan (2013) used the EPIC model to study yield gap for WW on the NCP, with an RMSE for biomass range between 1.18 and 2.0 Mg ha^−1^. Yu et al. (2006) calibrated and validated the Root Zone Water Quality Model (RZWQM) with both a generic plant growth module (RZWQM-G) and the CERES plant growth module (RZWQM-C) to simulate wheat-maize double cropping systems on the NCP and reported an RMSE of 2.07 Mg ha^−1^ with the RZWQM-G, and 2.26 t ha^−1^ with the RZWQM-C model. For simulated biomass, Heng et al. (2009) reported an RMSE range between 0.46 and 6.51 Mg ha^−1^ for maize using the AquaCrop model when using data from different locations. These results suggest that the CropSyst model provides a better simulation of above ground biomass than previous studies.

#### Grain yield

The deviation range of the current study validation was considerably better for grain yield (6.29 to −15.26%) for WW and SM from 2010–2011 to 2011–2012, than, for example, Araya et al. (2010) who report a deviation range of validation data of −13 to 15.1% for grain yield. Table 6 shows the statistical assessment of the CropSyst model for 3 experimental years. The results for RMSE (0.65 Mg ha^−1^), MAE (0.57 Mg ha^−1^), MBE (−0.08 Mg ha^−1^), d (0.82), and NMRSE (9.87%) of WW grain yield are comparable with those obtained by Singh et al. (2008) for WW grown at the Indian Agricultural Research Institute, New Delhi, using the same CropSyst model and CERES-Wheat for grain yield simulation. Wang et al. (2006) used CropSyst to simulate spring wheat with an RMSE of 13% of the observed means of grain yield in the Black Soil Zone of Northeast China. Rotation maize crop results with RMSE (0.2 Mg ha^−1^), MAE (0.13 Mg ha^−1^), MBE (−0.13 Mg ha^−1^), d (0.99), and NMRSE (3.79%) of grain yield are comparable with Stockle et al. (1997) who used a performance comparison study of sub-models (Penman Monteith-finite difference) of different levels of complexity in CropSyst. The results of the CropSyst model can be compared with other crop models used on the NCP for WW and SM yields. For example, Yu et al. (2006) calibrated and validated the Root Zone Water Quality Model (RZWQM) with both a generic plant growth module (RZWQM-G) and the CERES plant growth module (RZWQM-C) to simulate wheat and maize double cropping systems on the NCP; the overall simulation runs showed that the RZWQM-C model simulated grain yields with an RMSE of 0.94 Mg ha^−1^, compared to an RMSE of 1.23 Mg ha^−1^ with RZWQM-G. Fang et al. (2010) calibrated and validated RZWQM2, a hybrid model that combines the Root Zone Water Quality Model (RZWQM) and DSSAT4.0 to simulate wheat and maize grain yield with an RMSE of 0.59 Mg ha^−1^ and 0.71 Mg ha^−1^, respectively. Therefore, the results of this study suggest that the CropSyst model can be used with a considerable degree of accuracy to simulate the grain yield of WW and SM rotations in the NCP.

### ET_a_ partitioning into evaporation and transpiration for winter wheat 2008–2009

Simulated T closely matched observed T according to two different methods (i) micro-lysimeter subtracted from ET_a_ measured by EC (E(T)- MLS); (ii) the stable isotope method (E(T)-ISO). The deviation results of simulated vs. measured ratio T/ET_a_ in Table 8 are similar. The percentage comparison and statistical assessment of measured vs. simulated T/ET_a_ of the CropSyst model for WW 2008–2009, fell within an acceptable range and IoA was in the excellent range. E was 30% of total ET_a_ in the WW growing season of 2008–2009 and is comparable with results obtained by Zhang et al. (2011) for WW grown in LAEES, NCP.

### ET_a_ partition of wheat and maize crop rotation to identify evaporation losses

E accounts for 40% of total annual ET_a_ for the growing seasons of both WW and SM, as shown in Figure 6. These results are comparable with Liu et al. (2002) for WW and SM grown in LAEES, NCP. The pre-sowing irrigation method for wheat and maize is practiced in the NCP, in which irrigation is performed before harvesting of the previous crop and sowing is performed at soil field capacity. This leads to huge soil E losses after harvesting of the previous crop to emergence of the new crop up to the stage of canopy cover (see for instance, Figure 6).

Averages of the 3 years growth period for WW and SM were divided into 3 developmental periods; (i) PSI to EP, (ii) EP to CC (end of new leaf) period, and (iii) CC to HP, as shown in Table 9. Daily E accounts for ~10% of E in the pre-irrigation sowing to emergence period, ~60% emergence to canopy cover (end of new leaf) period, and ~30% canopy cover to harvesting period for both WW and SM crops. Most E occurs during the pre-sowing irrigation to canopy cover period. Therefore, results suggest that there is a need to reduce E through the use of precise irrigation (i.e., surface and sub-surface drip irrigation or by mulching) to overcome the huge water losses. For example, if we assume a precise irrigation method (such as drip irrigation), this saves 10% soil E losses (Evett et al., 2005; Abdelraouf and ElHabbasha, 2014; Qin et al., 2016). So, an irrigation reduction of 10% (ET_a_ reduction) across 1.7 million hectares of agricultural land in Hebei Province would account for approximately 1,700 million m^3^ year^−1^ of water saving.

**Table 9:** Evaporation losses with respect to growth stages.

**Growth periods**	**Wheat**		**Maize**	
	**Duration (days)**	**% of total evaporation**	**Duration (days)**	**% of total evaporation**
PES-EP	13.3	10.7	10.7	12.5
EP-CC	190.7	61.6	45.0	60.4
CC-HP	51.3	27.7	58.3	27.1

*PSI, Pre-sowing irrigation period; EP, emergence period; CC, canopy cover period; HP, harvesting period*.

The results of this study clearly indicate that the CropSyst model can be used with a high degree of accuracy for yield simulation and soil water loss analysis of WW and SM crop rotations on the NCP. It can also serve as a useful tool for assessing national food and water security in the agricultural sector.

## Summary and conclusions

Calibration of the CropSyst model (Suite-4) and its validation was tested in the 2010–2011 to 2012–2013 winter wheat (WW) and summer maize (SM) seasons in the arid and semiarid conditions of the NCP. Satisfactory agreements were obtained for ET_a_, biomass, and grain yield in the validation process. Major deviations were observed under conditions of severe stress. When the model was revalidated against WW (2008–2009), the results of the modeled ET_a_, biomass, and grain yield were well matched with a minimum deviation of 4.12%, −1.81, and −2.8%, respectively. In the same year, the simulated ET_a_ was split into evaporation (E) and transpiration (T), where, E was 30% of total ET_a_ and results closely matched the observed data collected during the MLM and isotopes approach. Based on the ET_a_ partition of WW (2008–2009), further ET partition of WW and SM from 2010–2011 to 2012–2013 into E and T showed that average evaporation was 40% of total ET_a_. E loss was high for two reasons, (i) pre-sowing irrigation practice is normally used in the NCP and sowing is performed at field capacity (ii) it is a result of flood irrigation. So, the gap between pre-sowing irrigation and canopy cover (end of new leaf stage) contribute toward a higher evaporation of ~70%. E losses from WW and SM (2010–2013) during the pre-sowing irrigation to the emergence period, the emergence to canopy cover period, and the canopy cover to harvesting period were ~10, 60, and 30%, respectively. Result reveals, there is high evaporation loss, so to reduce annual water deficit (P-ET) in typical irrigated croplands in NCP, it need to adopt advance methods (i.e., drip irrigation and mulching) to reduce E. We can conclude that the CropSyst model can be used with a reliable degree of accuracy to simulate crop rotations. This makes it a useful tool in the design and evaluation of deficit irrigation strategies that aim to prevent unnecessary loss from runoff, drainage, and soil evaporation, in addition to enhanced water-use efficiency.

## Author contributions

MU design the modeling study, perform the model analysis, synthesized results and constructed tables, figures for the paper and created the first draft. YS design the study and review the results. YQ collected weather and manage the field. YZ and HP collected crop data. AA contributed to review and final version of the manuscript. ML contributed to review the manuscript.

### Conflict of interest statement

The authors declare that the research was conducted in the absence of any commercial or financial relationships that could be construed as a potential conflict of interest.
